# Books

**Published:** 1889-12

**Authors:** 


### Books.

Pearson's Dentist's Appointment Book for 1890 is just out. This is a well arranged and very convenient little engagement book for the dentist's use. It can be carried conveniently in the vest pocket, or any other pocket, so that the practitioner may ever have with him a ready reference as to his appointments, and thus reduce to the minimum the liability of breaking engagements, for it is not always the patient that sins in this respect. Send to Pearson & Co., of Kansas City, and try the book.

